# Microbial culture vs. mNGS: diagnostic variations in periprosthetic joint infection

**DOI:** 10.3389/fcimb.2025.1611332

**Published:** 2025-10-28

**Authors:** Lan Lin, Xiaolin Li, Jiayu Li, Baijian Wu, Yiming Lin, Wenbo Li, Hongyan Li, Yufeng Guo, Chengguo Huang, Zida Huang, Wenming Zhang, Xinyu Fang

**Affiliations:** ^1^ Department of Orthopedics, First Affiliated Hospital of Fujian Medical University, Fuzhou, Fujian, China; ^2^ Department of Orthopedics, National Regional Medical Center, Binhai Campus of the First Affiliated Hospital, Fujian Medical University, Fuzhou, Fujian, China; ^3^ Fujian Provincial Institute of Orthopedics, The First Affiliated Hospital, Fujian Medical University, Fuzhou, Fujian, China; ^4^ Department of Orthopaedic Surgery, Changtai County Hospital, Zhangzhou, Fujian, China; ^5^ Department of Orthopaedic Surgery, Pingnan County Hospital, Ningde, Fujian, China

**Keywords:** periprosthetic joint infection, microbial culture, MNGs, pathogen identification, risk factors

## Abstract

**Objective:**

This study aimed to compare the diagnostic performance of conventional microbial culture and metagenomic next-generation sequencing (mNGS) in detecting pathogens in periprosthetic joint infection (PJI) and to identify factors contributing to discrepancies between these two methods.

**Methods:**

A total of 167 patients with suspected PJI (including PJI patients and aseptic failure patients) who underwent revision joint replacement at our center from September 2017 to April 2024 were enrolled. Demographic data, prior antibiotic use, and results of microbial culture and mNGS were documented. Joint fluid, periprosthetic tissue, or prosthetic ultrasonic fluid samples were collected, and at least one sample from each patient underwent both microbial culture and mNGS testing. In the light of the concordance between culture and mNGS results, patients were divided into the detection consistent and detection inconsistent groups. The differences in pathogen detection between the two models were compared, and factors contributing to discordant results were analyzed.

**Results:**

The prior antibiotic use (OR = 2.137, 95% CI = 1.069-4.272, *P* = 0.032), polymicrobial infections (OR = 3.245, 95% CI = 1.278-8.243, *P* = 0.013), infection caused by rare pathogens (OR = 2.735, 95% CI = 1.129-6.627, *P* = 0.026), and intraoperative tissue specimens (OR = 2.837, 95% CI = 1.007-7.994, *P* = 0.049) were identified as risk factors for discordance between microbial culture and mNGS results, particularly in cases with negative microbial culture but positive mNGS findings. Conversely, consistency in specimen type (OR = 0.471, 95%CI=0.254-0.875, *P* = 0.017) was identified as a protective factor against discordance.

**Conclusion:**

Clinicians should optimize diagnostic strategies by tailoring microbial culture methods to the patient’s clinical condition and integrating mNGS testing where appropriate. It is recommended to use tissue specimens from the same anatomical site across multiple tests while sampling from different regions when necessary. Although this approach may increase costs, it significantly enhances the accuracy of pathogen identification and facilitates more effective treatment.

## 1 Introduction

Joint replacement surgery is performed on millions of patients globally each year to alleviate pain, improve joint function, and enhance overall quality of life. Despite its benefits, periprosthetic joint infection (PJI) remains a serious complication, posing a major challenge in both primary and revision joint replacement procedures. PJI has an incidence rate ranging from 0.5% to 7.0% and is linked to a high mortality rate of 2.7% to 18.0% (Bozic et al., 2010; Kurtz et al., 2010). The occurrence of PJI can have devastating impacts on patients’ health and impose substantial economic burdens on individuals and healthcare systems (Bozic et al., 2010; Kurtz et al., 2010; Illingworth et al., 2013).

Timely and accurate identification of the pathogens responsible for PJI is critical for effective diagnosis and treatment. Currently, microbial culture is widely regarded as the “gold standard” for pathogen detection in PJI cases. Culturing bacteria and conducting targeted antimicrobial therapy remain essential for maximizing treatment success (Yang et al., 2020). However, 20% to 50% of patients with clear clinical and laboratory evidence of PJI exhibit negative culture results (Parvizi et al., 2006; Berbari et al., 2007; Parvizi et al., 2014). Several factors contribute to the limited sensitivity of culture-based methods in identifying pathogenic microorganisms, including insufficient sample size, prior antibiotic exposure, suboptimal culture techniques, and the unique biological characteristics of certain pathogens (Parvizi et al., 2014; Abdel et al., 2019; Amanatullah et al., 2019). This diagnostic challenge, often referred to as “culture-negative-PJI,” can significantly hinder clinical decision-making and compromise treatment outcomes (Tan et al., 2018; Kim and Cho, 2021). As a result, there is growing interest in exploring alternative diagnostic approaches that can improve pathogen detection in PJI cases.

Next-generation sequencing (NGS) has seen continuous development and has been widely applied in clinical diagnostics (Gu et al., 2019). Among its applications, metagenomic NGS (mNGS) is the most extensively studied. As a culture-independent microbial molecular diagnostic technology, mNGS combines high-throughput sequencing with bioinformatics analysis to identify the types and abundance of all known microorganisms within a sample (Fang et al., 2020). Studies have demonstrated the significant value of mNGS in diagnosing pathogens associated with various infectious diseases (O’Flaherty et al., 2018; Chen et al., 2022). In particular, mNGS has shown high sensitivity in diagnosing PJI (Ivy et al., 2018; Fang et al., 2020), especially in cases of “culture-negative PJI” (Wang et al., 2020). However, the unbiased detection of all nucleic acid fragments by mNGS frequently results in false positives, which can complicate clinical decision-making (Han et al., 2022; Liu et al., 2022; Rimoldi et al., 2023).

Discrepancies between the traditional microbial culture and mNGS results often challenge clinicians in accurately identifying the causative pathogens of PJI. This uncertainty can hinder the appropriate selection of targeted antibiotics, potentially leading to ineffective treatment and, ultimately, failure of PJI management. While existing literature primarily highlights the advantages of mNGS in diagnosis (Mei et al., 2023), there is limited discussion on the differences between culture-based and mNGS-based diagnostic methods. To address this gap, the present study focuses on comparing the diagnostic discrepancies between these two methods. It also seeks to analyze the underlying reasons for these differences, with the aim of identifying strategies to improve the accuracy of etiological diagnosis.

## 2 Methods

### 2.1 Patient selection

This cohort study was granted by the Ethics Committee of the First Affiliated Hospital of Fujian Medical University (MRCTA, FMU ECFAH [2015]084-2). We enrolled patients suspected of having PJI who underwent revision arthroplasty at our institution between September 2017 and April 2024. The inclusion criteria were: (1) patients diagnosed with suspected PJI, as determined by medical history, physical examination, and auxiliary diagnostic tests; (2) at least one specimen from preoperative joint fluid aspiration, intraoperative joint fluid, periprosthetic tissue, or prosthetic ultrasonic fluid was cultured and tested for pathogens using both conventional microbiological methods and mNGS; and (3) complete case data. Exclusion criteria included (1) incomplete clinical records or an unclear diagnosis and (2) evident contamination during the sampling process. The diagnosis of PJI was independently reviewed by a panel of at least two senior orthopedic surgeons, two senior infectious disease specialists, and one senior microbiologist, following the diagnostic criteria outlined by the Musculoskeletal System Infection Association (MSIS) (Parvizi et al., 2011).

### 2.2 Sampling operation

Intraoperative joint fluid collection: puncture was performed prior to the incision of the joint capsule to minimize blood contamination. Once the joint fluid is drawn, it is immediately injected into aerobic, anaerobic, or fungal culture bottles for subsequent microbial analysis.

Prosthesis ultrasound fluid collection: sterile transport containers, such as sealed boxes, centrifuge tubes, etc., were prepared through plasma sterilization for future use. During surgery, removed prosthetic components (such as knee joint pads, hip joint liners, etc.) were placed in 400 ml of sterile saline and subjected to ultrasonic treatment (40 Hz, 5 min) to disrupt the biofilm. The ultrasonic lysate was then centrifuged, and the supernatant was carefully separated for microbial culture.

Sampling of periprosthetic tissues: using a sterile scalpel, periprosthetic tissue samples were excised from 3 different sites exhibiting inflammation, ensuring no contamination with joint fluid. These samples were placed into sterile containers for processing, including digestion, grinding, and subsequent microbial culture.

### 2.3 Specimen collection and microbial culture

Fluid specimens (e.g., joint fluid, prosthetic ultrasonic fluid, etc.) were transferred into Bactec Plus/F or BactecPeds Plus/F aerobic and anaerobic blood culture bottles (Becton Dickinson, Germany). Cultures were maintained in a Bactec 9050 automated thermostat (Becton-Dickinson) at 35-37°C with 5-7% CO_2_. Aerobic cultures were incubated for 5–6 days, and anaerobic cultures for 10–14 days. The periprosthetic tissue was minced and digested utilizing 1 ml of trypsin (Qingdao Haibo Biotechnology Co., Ltd., HBPM0153) in an automatic grinder (40 Hz, 90 s) until homogenized. The resulting tissue homogenate was plated onto blood culture plates, followed by culturing under anaerobic and aerobic conditions, with culture conditions identical to those for liquid specimens. Microbial identification was performed employing MALDI-TOF mass spectrometry and the VITEKII biochemical identification system, which also includes antimicrobial susceptibility testing. A positive culture from joint fluid or periprosthetic tissue samples was determined by the detection of the same microorganism in at least two independent joint fluids or tissue samples. A positive culture from prosthetic ultrasonic lysate was determined by the identification of highly virulent pathogens such as *Staphylococcus aureus*, *Escherichia coli*, and *Streptococcus*, or low-virulence pathogens such as *coagulase-negative Staphylococci* and *Corynebacterium*, with a colony count >50 colony-forming units (CFU)/culture plate.

### 2.4 mNGS

The specimen pretreatment procedures were consistent with those used for microbiological culture, and the mNGS detection protocol has been previously described (Fang et al., 2021). Briefly, the procedure involved the following steps: (1) Total DNA was extracted from synovial fluid, sonicated fluid, or homogenized tissue utilizing the TIANamp Micro DNA Kit (DP316, Tianjin, China) by cell wall disruption. (2) The extracted DNA was sonicated to create 200-300-bp fragments, followed by PCR amplification and circularization to create DNA nanospheres. These were then sequenced on the BGI SEQ-500 platform (UWIC, China). To ensure accuracy, negative controls (double-distilled water) were included in each batch. If contamination was highly suspected, samples were reprocessed starting from nucleic acid extraction. Common contamination scenarios typically included the detection of pathogens in the negative control samples or a simultaneous high number of the same pathogen across most samples in a batch. (3) The raw sequencing data were processed utilizing a bioinformatics pipeline developed by BGI. The human reference genome sequence (Hg19) was removed by Burrows-Wheeler alignment, and the resultant sequences were compared against an internal microbial genome database established by BGI to identify species of bacteria, fungi, and viruses. (4) Potential pathogens were distinguished from background microorganisms based on their relative abundance and the number of reads. Based on established criteria in the literature (Ding et al., 2024), the following thresholds were applied: 1. For common background microorganisms (e.g., Burkholderia spp, Delftia spp, Sphingomonas spp, Streptomyces spp, and Albugus spp), a genus-level relative abundance of ≥80% was required for pathogen identification. 2. Within pathogenic genera, the species demonstrating the highest genome coverage rate and standardized number of reads stringently mapped at the species level (SDSMRNS) was designated as the causative pathogen. 3. For the Mycobacterium tuberculosis complex, which typically yields minimal nucleic acid, the standardized number of reads stringently mapped at the genus level (SDSMRNG) served as the primary diagnostic criterion.

### 2.5 Methods for determining the reliability of microbiological results

(1) The pathogen was considered to cause periprosthetic joint infection if it had been previously identified in the literature as a causative agent and was consistent with the patient’s clinical characteristics. (2) Targeted microbial therapy was considered effective if at least three senior clinicians independently confirmed the diagnosis and the therapeutic outcome.

### 2.6 Statistical analysis

Difference comparison between the two groups was made utilizing the chi-square test or Fisher’s exact test. McNemar’s test (two-tailed) was adopted to assess the differences in sensitivity and specificity between the two diagnostic methods. Continuous variables with a normal distribution are summarized as the mean ± standard deviation, while count data are reported as numbers (percentages). All analyses were completed employing SPSS software (version 26.0, IBM, USA). *P*<0.05 signified statistical significance.

## 3 Results

### 3.1 Microbial culture and mNGS results for suspected PJIs

**Figure 1:**
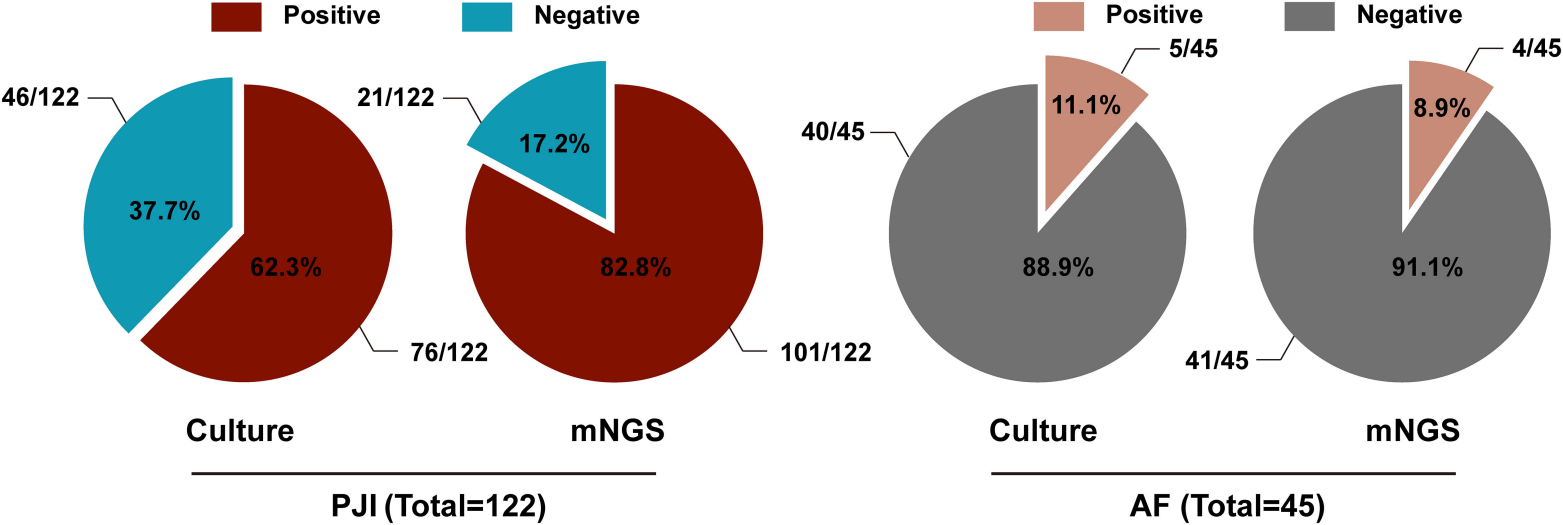
The proportion of microbiological culture and mNGS results in the PJI group and the AF group.

Totally, 175 patients were screened by referring to the inclusion criteria, and 8 cases were excluded (due to severe sample contamination or ambiguous diagnosis). Ultimately, 167 patients were included in the study. In the light of the diagnostic criteria for PJI as described by MSIS (Parvizi et al., 2011), 122 cases were diagnosed with PJI and 45 cases with aseptic failure (AF). In the PJI group, microbiological culture was positive in 76 cases (76/122, 62.3%), while mNGS was positive in 101 cases (101/122, 82.8%). In contrast, in the AF group, 5 cases were positive by microbiological culture (5/45, 11.1%), and 4 cases were positive by mNGS (4/45, 8.9%) (**Figure 1**). The diagnostic performance of both tests is summarized in **Table 1**. The microbiological culture method exhibited a sensitivity of 62.3% and a specificity of 88.9%. Its positive predictive value (PPV) was 93.8%, and the negative predictive value (NPV) was 46.5%, yielding an overall accuracy of 69.5%. In contrast, the mNGS assay exhibited a sensitivity of 82.8%, a specificity of 91.1%, a PPV of 96.2%, and an NPV of 66.1%, resulting in an accuracy of 85.0%. Notably, mNGS demonstrated a significantly higher sensitivity than microbiological culture (*p* = 0.001). Although mNGS showed a higher specificity, this difference was not statistically significant (*p* = 0.681).

**Table 1:** Comparison of diagnostic efficiency between microbial culture and mNGS.

Method	PJI group (n=122)	AF group (n=45)	Sensitivity (%)	Specificity (%)	PPV (%)	NPV (%)	Accuracy (%)
Culture positive	76	5	62.3%	88.9%	93.8%	46.5%	69.5%
mNGS positive	101	4	82.8%	91.1%	96.2%	66.1%	85.0%
P-value	/	/	0.001*	0.681	/	/	/

PPV, positive predictive value; NPV, negative predictive value.

### 3.2 Comparison of results between microbial culture and mNGS in suspected PJIs

Among the 122 patients in the PJI group, 63 (51.6%) had positive results for both tests. In 36 cases (29.5%), the pathogen test results were fully compatible between the two methods, while in 10 cases (8.2%), the results were partially compatible. There were 17 cases (13.9%) in which both tests were positive, but the results were completely inconsistent, and 13 cases (10.7%) in which mNGS was negative and the culture was positive. Additionally, 38 cases (31.1%) had a positive result with mNGS but a negative result (**Supplementary Table 1**). Of the 36 patients with identical positive results, clinicians achieved favorable therapeutic outcomes after treatment with sensitive antibiotics, with a 100% success rate. Of the 10 patients with partially consistent double-positive results, 9 experienced good treatment outcomes, and 1 relapsed, resulting in a treatment success rate of 90.0%. In the 17 PJI cases with completely inconsistent positive results between the two tests, 14 cases achieved good outcomes, and 3 cases relapsed after treatment with antibiotics covering both pathogens, yielding a success rate of 88.2%. In the 13 PJI cases with negative mNGS and positive culture, all patients responded well to antibiotic treatment, with a 100% treatment success rate. In contrast, among 38 PJI cases with positive mNGS and negative culture, 34 achieved favorable outcomes, and 4 relapsed, giving a treatment success rate of 89.5%. In the AF group (n = 45), 4 patients (8.9%) had positive results by both tests, 1 patient (2.2%) had a positive culture but negative mNGS, and no patients (0%) had a positive mNGS but negative culture results (**Supplementary Table 1**).

### 3.3 Risk factors for discrepancies between microbial culture and mNGS

Patients were divided into two groups by referring to whether the results from microbial culture and mNGS were consistent: the detection consistent (DC, n = 88, excluding those with positive results from both tests but partially consistent pathogen identification) and detection inconsistent (DI, n = 79) groups. No significant differences in age, gender, BMI, or disease location were noted between the two groups (**Table 2**). Notably, 9 patients in the DC group and 22 in the DI group had multiple infections, with this difference being statistically significant (*P* = 0.003) (**Table 2**). Most rare microorganisms, such as *Mycoplasma*, *non-tuberculous mycobacteria*, and *Parvimonas micra*, require special culture conditions and are often undetectable by conventional clinical microbiology laboratories (Thoendel et al., 2017; Forbes et al., 2018; Chen et al., 2020). In the current study, 10 patients in the DC group and 24 patients in the DI group were infected with rare pathogens, a difference that was statistically significant (*P* = 0.002) (**Table 2**). Moreover, a larger proportion of patients in the DI group (39/79, 49.4%) had received antibiotic treatment prior to sampling compared to those in the DC group (28/88, 31.8%) (*P* = 0.021) (**Table 2**). The specimens used for microbiological culture and mNGS were also distributed differently between the two groups: 106 specimens were collected from the DC group (45 joint fluid, 30 intraoperative tissue, and 31 prosthesis ultrasound fluid), while 101 specimens were collected from the DI group (28 joint fluid, 46 intraoperative tissue, and 27 prosthesis ultrasound fluid). The distribution of specimen types between the two groups was statistically different (*P* = 0.024) (**Table 2**). In the DC group, 52 patients had concordant specimen types for both tests, significantly more than the DI group, where concordance was less common (*P* = 0.016) (**Table 2**). In summary, factors such as prior antibiotic use, multiple infections, rare pathogen infections, specimen type, and specimen concordance may contribute to discrepancies between microbial culture and mNGS results.

**Table 2:** Characteristics of patients.

Characteristics	DC group (n=88)	DI group (n=79)	P-value
Age, median years (range)	65.3 ± 11.8	63.1 ± 14.0	0.481
Sex, female%	45(51.5%)	41(51.9%)	0.922
BMI(kg/m^2^)	24.1 ± 2.9	25.4 ± 3.1	0.470
Joints			0.086
Hip	51	40	
Knee	37	35	
Elbow	0	4	
Multiple infections	9	22	0.003
Infections with rare pathogens	10	24	0.002
Use antibiotics before sampling	28	39	0.021
Total specimen	106	101	0.024
Joint fluid	45	28	
Intraoperative tissue	30	46	
Prosthesis ultrasound fluid	31	27	
Consistency of test samples	52	32	0.016

DC group, Detection consistent group; DI group, Detection inconsistent group; BMI, Body mass index.

**Figure 2:**
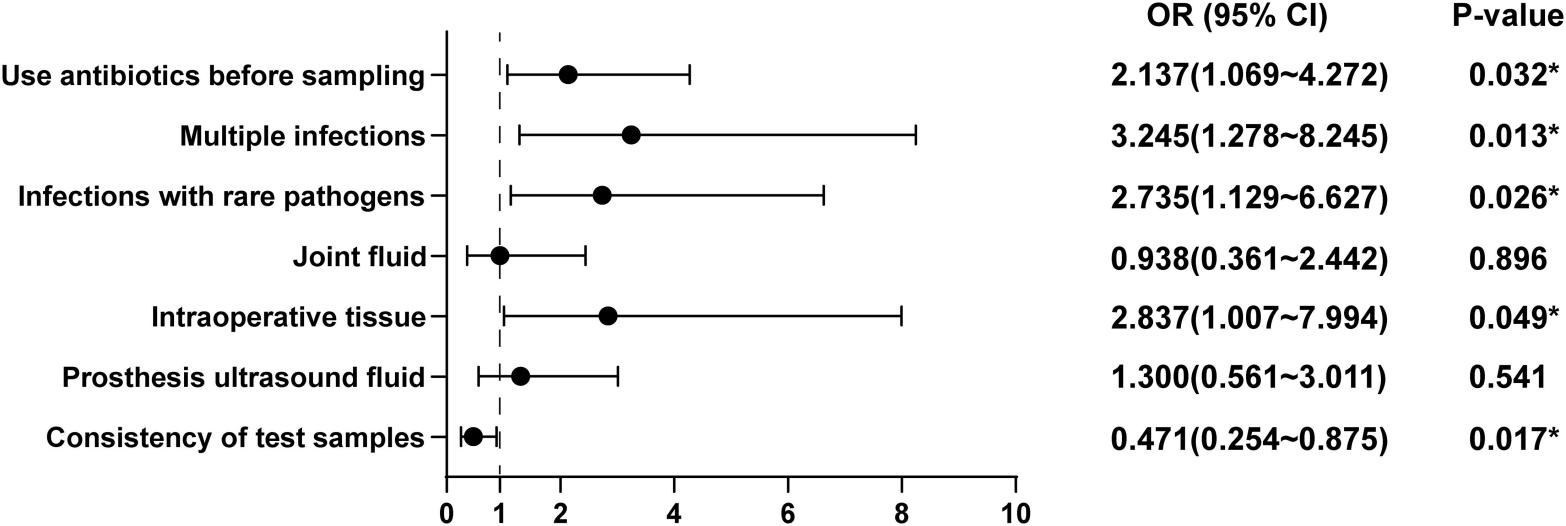
Risk factors for discrepancies between microbial culture and mNGS.

To further explore the risk factors contributing to discrepancies in test results, we constructed a multivariate logistic regression model, including variables such as antibiotic use before sampling, multiple infections, rare bacterial infections, specimen types, and specimen concordance. The results of the model, shown in **Figure 2**, indicated several significant findings: The risk of inconsistency between the two tests was 2.137 times higher in patients who had received antibiotics prior to sampling compared to those who had not (OR = 2.137, 95% CI = 1.069-4.272, *P* = 0.032). Patients with multiple infections had 3.245 times higher odds of having inconsistent results between the two tests compared to those without multiple infections (OR = 3.245, 95% CI = 1.278-8.243, *P* = 0.013). The risk of inconsistent results was 2.735 times higher in patients infected with rare pathogen infections compared to those without such infections (OR = 2.735, 95% CI = 1.129-6.627, *P* = 0.026). Intraoperative tissue samples were linked to a 2.837-fold increase in the risk of test result discordance compared to non-intraoperative tissue samples (OR = 2.837, 95% CI = 1.007-7.994, *P* = 0.049). However, no increased risk was noted when joint fluid (OR = 0.938, 95% CI = 0.361-2.442, *P* = 0.896) or prosthesis ultrasound fluid (OR = 1.300, 95% CI = 0.561-3.011, *P* = 0.541) was used as a specimen. Concordance between the specimens used for microbiological culture and mNGS was found to be a protective factor, with patients having concordant test specimens experiencing a 52.9% lower risk of discrepancies between the two tests relative to those with discordant specimens (OR = 0.471, 95% CI = 0.254-0.875, *P* = 0.017).

### 3.4 Impact of antibiotic use, multiple infections, rare pathogens infections, and specimen type on microbial culture results

To further explore the specific factors influencing discrepancies in test results, the DI group was divided into 3 subgroups in the light of the patterns of microbial culture and mNGS results: Culture positive-mNGS negative, Culture negative-mNGS positive, and Culture positive-mNGS positive*(both tests positive but with different pathogen results. The distribution of factors such as antibiotic use, multiple infections, rare pathogen infections, and specimen type for each of these subgroups is detailed in **Table 3**. Among the Culture negative-mNGS positive subgroup, 26 cases (68.4%) had received antibiotics before sampling. This proportion was notably higher than that in the Culture positive-mNGS negative (28.6%) and Culture positive-mNGS positive* groups (33.3%) (*P* = 0.005). In terms of multiple infections, 13 patients (34.2%) in the Culture negative-mNGS positive group had multiple infections, which was a higher proportion compared to the other two subgroups. However, the difference did not achieve statistical significance (*P* = 0.256). Similarly, rare pathogen infections were observed in 14 patients (36.8%) in the Culture negative-mNGS positive group, a higher proportion than in the other two groups, but again, this difference was not statistically significant (*P* = 0.445). Regarding specimen type, the proportions of joint fluid (*P* = 0.716) and prosthesis ultrasound fluid (*P* = 0.699) did not differ significantly between the three groups. However, a statistically significant difference was observed for intraoperative tissue. A total of 29 patients (76.3%) in the Culture negative-mNGS positive group had intraoperative tissue as the specimen, which was significantly higher than the proportion in the Culture positive-mNGS negative group (35.7%) and the Culture positive-mNGS positive* group (44.4%) (*P* = 0.006). These findings suggest that the factors contributing to discrepancies between microbial culture and mNGS results, such as prior antibiotic use, multiple infections, rare pathogen infections, and the use of intraoperative tissue as a specimen, are primarily concentrated in patients with negative microbial culture but positive mNGS findings.

**Table 3:** Specific antibiotic use, multiple infections, rare pathogens infections, and specimen type distribution of patients with different results.

Characteristics	Culture positive mNGS negative (n=14)	Culture negative mNGS positive (n=38)	Culture positive mNGS positive* (n=27)	P-value
Use antibiotics before sampling	4 (28.6%)	26 (68.4%)	9 (33.3%)	0.005*
Multiple infections	2 (14.3%)	13 (34.2%)	7 (25.9%)	0.256
Infections with rare pathogens	4 (28.6%)	14 (36.8%)	6 (22.2%)	0.445
Joint fluid	5 (35.7%)	15 (39.5%)	8 (29.6%)	0.716
Intraoperative tissue	5 (35.7%)	29 (76.3%)	12 (44.4%)	0.006*
Prosthesis ultrasound fluid	6 (42.9%)	13 (34.2%)	8 (29.6%)	0.699

*: Patients with both positive tests and non-identical and completely different results.

### 3.5 Frequency distribution of microorganisms with different detection results between microbial culture and mNGS in suspected PJIs

**Figure 3:**
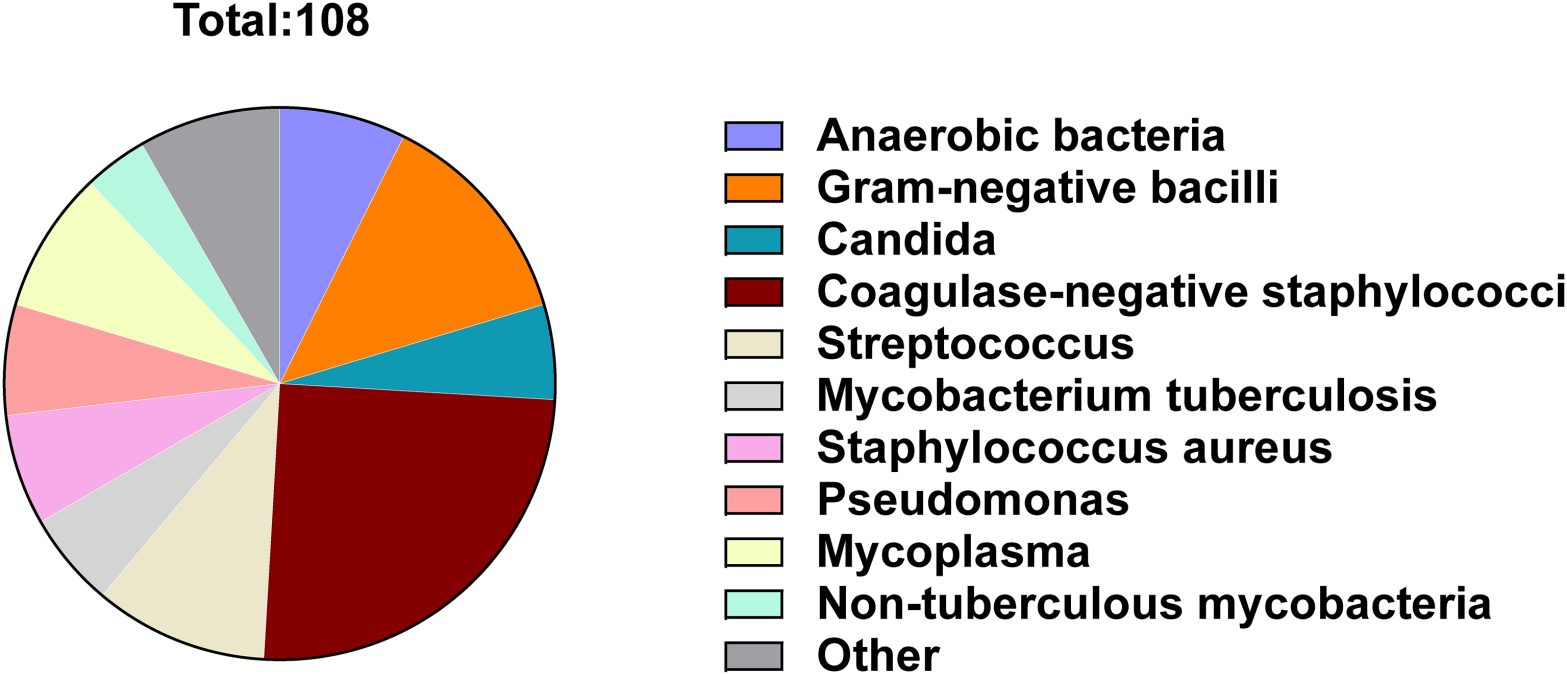
Frequency distribution of microorganisms with different results detected by microbial culture and mNGS.

The differences in microorganism detection results between microbial culture and mNGS were summarized for the PJI and AF groups (**Figure 3**). A total of 108 microorganisms showed discrepancies between the two tests. The microorganisms with the most frequent discrepancies were primarily *Coagulase-negative staphylococci* (27, 25.0%), *Gram-negative bacilli* (14, 13.0%), *Streptococcus* (11, 10.2%) and *Mycoplasma* (9, 8.3%) (**Figure 3**). Among the 45 cases of differential microorganisms between the two tests in patients with both positive microbiological cultures and mNGS findings, *Coagulase-negative staphylococci* were most prevalent (13, 28.9%). In contrast, 17 microorganisms were detected with positive microbial cultures but negative mNGS findings, with *Coagulase-negative staphylococci* being the most common (8, 47.1%). Conversely, 46 microorganisms were detected with negative microbial cultures but positive mNGS findings, with *Streptococcus* (9, 19.7%) and *Mycoplasma* (9, 19.7%) being the predominant organisms. When comparing patients with positive microbial culture but negative mNGS results to those with negative microbial culture but positive mNGS results, the latter group had distinctly more detections of *Streptococcus* (*P* = 0.047) and *Mycoplasma* (*P* = 0.047), while fewer *Coagulase-negative staphylococci* (P = 0.004) were detected (**Table 4**).

**Table 4:** Distribution of pathogens with inconsistent results between microbial culture and mNGS testing in the PJI and AF groups.

Pathogen	Culture positive, mNGS negative (n=17)	Culture negative, mNGS positive (n=46)	P-value
*Anaerobic bacteria*	1	5	0.481
*Gram-negative bacilli*	2	6	0.631
*Candida*	1	1	0.470
*Coagulase-negative staphylococci*	8	5	0.004
*Streptococcus*	0	9	0.047
*Mycobacterium tuberculosis*	0	2	0.530
*Staphylococcus aureus*	1	3	0.707
*Pseudomonas*	1	2	0.618
*Mycoplasma*	0	9	0.047
*Non-tuberculous mycobacteria*	1	3	0.707
*Other*	2	1	0.175

## 4 Discussion

The use of antibiotics targeting pathogenic microorganisms is a critical strategy for treating PJI. Therefore, early and accurate pathogen identification is critical for effective treatment. Although microbial culture has been a cornerstone in diagnosing PJI, its sensitivity and accuracy often fall short of clinical requirements. Recently, mNGS has highlighted as a valuable diagnostic tool due to its high sensitivity and its ability to complement microbial culture in pathogen detection (Mouraviev and McDonald, 2018; Chen et al., 2022). However, discrepancies between the results of microbial culture and mNGS can pose significant challenges for clinicians, potentially delaying or even hindering proper treatment. To explore the reasons behind these discrepancies, microbial culture and mNGS test data were gathered from 167 patients suspected of PJI at our center. Our findings suggested that several factors contributed to differences between the results of the two diagnostic methods. These include prior antibiotic use, multiple infections, rare pathogen infections, and the use of intraoperative tissue as a specimen. Additionally, we found that ensuring the consistency of test specimens could reduce the likelihood of discrepancies between the two methods.

Most PJI patients receive broad-spectrum antibiotic therapy before hospital admission to reduce bacterial load to some extent. However, this practice can affect the accuracy of pathogen detection. Previous studies have highlighted that prior antibiotic use is a significant risk factor for negative microbial culture results (Berbari et al., 2007; Kalbian et al., 2020). In our study, we observed that among patients who used antibiotics before sampling and had discrepancies between microbial culture and mNGS, the majority (26/39, 66.7%) had negative microbial cultures but positive mNGS findings. This suggests that microbial culture is more sensitive to the effect of antibiotics, which can easily lead to negative cultures, whereas mNGS is more resistant to antibiotic interference. The 2018 International Consensus Meeting (ICM) on PJI emphasized the importance of avoiding antibiotics before a definitive diagnosis of PJI (Schwarz et al., 2019). Therefore, to reduce the risk of negative culture findings and improve diagnostic accuracy, broad-spectrum antibiotics should be withheld until a clear diagnosis is made.

The presence of multiple infections can significantly affect the accuracy of pathogen diagnosis (Hoffman et al., 2006). In the present study, 22 patients with discrepant results between the two tests had mixed infections (22/79, 27.8%). These mixed infections were primarily concentrated in patients with negative microbial cultures but positive mNGS findings (13/22, 59.1%). Previous research has documented that when multiple microorganisms are present at the infection site, species competition can occur (Hoffman et al., 2006), with dominant pathogens suppressing the growth of others. As the microbial flora becomes more complex, the likelihood of obtaining a negative culture increases. In contrast, mNGS can rapidly and comprehensively sequence the genetic material of all microorganisms in a sample, making it particularly valuable for diagnosing mixed infections compared to traditional microbial culture (Xie et al., 2023).

In this study, pathogens identified in patients with positive microbial cultures but negative mNGS findings were more commonly *Coagulase-negative staphylococci* than those in patients with negative microbial cultures but positive mNGS findings (*P* = 0.004). The thick cell wall of *Coagulase-negative staphylococci* makes it difficult to break during nucleic acid extraction, which can reduce DNA extraction efficiency (Shi et al., 2024). If the cell wall disruption process is insufficient, DNA from the bacteria may not be effectively extracted, resulting in a low number of detected sequences and potentially leading to a negative mNGS result. Conversely, *Streptococcus* (*P* = 0.047) and *Mycoplasma* (*P* = 0.047) were more commonly found in patients with negative microbial cultures but positive mNGS results. *Streptococcus* has relatively demanding culture conditions, as it requires CO_2_ for optimal growth. Some species, such as *Streptococcus viridans*, are facultative anaerobes that grow better in a 5%-10% CO_2_ environment (Pulliam et al., 1980). Additionally, *Streptococci* are sensitive to environmental changes such as drying and temperature changes, which may cause them to enter a viable but non-culturable state in adverse conditions (Fakruddin et al., 2013). Moreover, during sampling, some *Streptococci* can be masked by other fast-growing species (Weng et al., 2022). Mycoplasma, the smallest prokaryotic organism, is a common pathogen of urogenital tract infection. However, mycoplasma requires special culture medium to produce positive results, and mNGS has good sensitivity for the detection of mycoplasma (Wang et al., 2021; Cai et al., 2023).

The discrepancy in test results for intraoperative tissue specimens may be related to the variation in tissue locations within the body. Different tissues and organs are highly specialized in their functions, and this differentiation can influence pathogen detection. For example, the synovium, joint capsule, and synovial fluid each play distinct roles within the joint cavity, contributing to complex physiological processes. Studies have shown that drug concentration levels can vary greatly between different tissues in the joint (Hermsen et al., 2012). Depending on the method of administration and the tissue penetration of the drug, these differences in drug concentrations may influence the bacterial colonization sites, thereby increasing the likelihood of detecting different pathogens in various tissue locations. In the case of prosthetic ultrasonic fluid and joint fluid, the sampling procedure was identical for both fluids, and they were mixed during the collection process. As a result, pathogens were evenly distributed across the fluid, which may explain the minimal differences in results between the two tests. In our study, the consistency of specimen type was shown to be a protective factor against discrepancies in test results. For the three specimen types—tissue, prosthetic ultrasonic fluid, and joint fluid—the test outcomes were closely linked to the characteristics and distribution of microorganisms. This consistency in specimen type also contributed to the alignment of test results by reducing variability in specimen collection and processing.

In clinical practice, it is challenging to use the same sample for both pathogen culture and mNGS testing due to the differing specimen processing requirements, limitations in specimen volume, the heterogeneity of infected areas, and the independence of the two testing procedures. These factors make it difficult to achieve complete consistency in sample testing during routine operations. While this presents certain challenges, it also offers some advantages. The complexity of PJI lies in the diversity of its pathogens, which necessitates the use of multiple specimen types to comprehensively identify pathogens, ensure broad coverage for antibiotic treatment, and avoid missing potential sources of infection. However, this multi-sample strategy increases the risk of contamination, which can complicate diagnostic and treatment decisions for clinicians. Therefore, it is recommended to use samples from the same anatomical site for multiple testing methods, while also ensuring coverage of different anatomical sites when possible. Although this approach may increase diagnostic costs, it can provide more accurate pathogen information, help develop better treatment plans, and ultimately improve both treatment and patient outcomes.

Despite its high sensitivity, the unbiased nature of mNGS necessitates a critical discussion of false-positive results. In our cohort, the specificity of mNGS was 91.1%, with 4 out of 45 (8.9%) aseptic failure (AF) patients yielding a positive mNGS result. These findings could be attributed to several factors. First, the detection of low-level environmental contaminants (e.g., Burkholderia spp, Delftia spp, or Sphingomonas spp) introduced during sample collection or laboratory processing is a well-known challenge. Second, mNGS can detect non-viable microbial nucleic acids from prior, resolved infections or from the perioperative environment, which do not represent active, clinically relevant infection (Rimoldi et al., 2023). To mitigate these issues, we implemented stringent bioinformatic filters, as described in the Methods, including thresholds for relative abundance and the use of standardized read counts (SDSMRNS/SDSMRNG) to distinguish true pathogens from background noise (Ding et al., 2024). Furthermore, the clinical context and the MSIS diagnostic criteria remained the ultimate arbiters in differentiating contamination from true infection. Therefore, while a positive mNGS result is highly informative, it must be interpreted cautiously in conjunction with clinical and laboratory findings.

Beyond elucidating diagnostic discrepancies, our findings underscore the clinical value of integrating mNGS into the PJI workflow. Its enhanced sensitivity directly informed clinical decisions, especially in cases with prior antibiotic use, polymicrobial, or rare pathogen infections. For the 38 culture-negative but mNGS-positive PJI patients, results prompted empirical therapy modifications in all cases, guiding targeted regimens—such as administering macrolides upon detecting culture-missed *Mycoplasma*. This shift from broad-spectrum to pathogen-directed therapy optimizes efficacy and mitigates resistance risks. Moreover, mNGS’s rapid turnaround significantly shortened time to targeted therapy compared to prolonged cultures for anaerobes or fungi, a critical acceleration since treatment delays correlate with failure (Tan et al., 2018). Although a formal cost-analysis was beyond our scope, the high success rate (89.5%, 34/38) in this subgroup indicates a positive patient outcome impact. By revealing otherwise undetected pathogens, mNGS enabled definitive strategies, potentially reducing revision surgeries, hospital stays, and improving joint function. Future studies with cost-effectiveness and patient-reported metrics are needed to fully quantify these benefits.

This study still has several potential limitations. First, as a single-center study, our relatively small sample size (n=167) may affect statistical power and result reliability, particularly in subgroup analyses (e.g., rare pathogens or specific specimen types). Second, the retrospective design could not fully control for confounding factors, such as variations in pre-sampling antibiotic administration strategies across different periods. Third, although PJI diagnoses were based on established MSIS criteria and expert consensus, we did not quantify the interobserver agreement using a Kappa statistic. Finally, the study did not include comparative analyses with other emerging technologies (e.g., targeted sequencing or 16S PCR). Future multicenter, prospective studies with larger sample sizes incorporating multiple molecular diagnostic techniques are needed to further validate the generalizability of our findings.

## 5 Conclusions

This study identified several key risk factors for discrepancies between microbial culture and mNGS results, including prior antibiotic use, multiple infections, rare pathogen infections, and the selection of tissue specimens during surgery. In contrast, the consistency in test specimen types served as a protective factor against discrepancies. The pathogens with the most notable detection differences between the two tests were primarily *Coagulase-negative staphylococci*, *Gram-negative bacilli*, *Mycoplasma* and *Streptococcus.* PJI is a complex infectious disease, and while multi-sample testing is essential for thorough pathogen identification and comprehensive diagnosis, it also increases the risk of contamination, which can affect clinicians’ treatment options. Therefore, it is recommended to use tissue specimens from the same site for multiple tests, covering different anatomical sites at the same time. Although this may incur higher costs, it provides more accurate pathogen information and facilitates more effective treatment.

## Data Availability

The original contributions presented in the study are included in the article/**Supplementary Material**. Further inquiries can be directed to the corresponding authors.

## References

[B1] Abdel M. P. Akgün D. Akin G. Akinola B. Alencar P. (2019). Hip and knee section, diagnosis, pathogen isolation, culture: proceedings of international consensus on orthopedic infections. J. Arthroplasty. 34, S361–s367. doi: 10.1016/j.arth.2018.09.020, PMID: 30343972

[B2] Amanatullah D. Dennis D. Oltra E. G. Marcelino Gomes L. S. Goodman S. B. (2019). Hip and knee section, diagnosis, definitions: proceedings of international consensus on orthopedic infections. J. Arthroplasty. 34, S329–s337. doi: 10.1016/j.arth.2018.09.044, PMID: 30348576

[B3] Berbari E. F. Marculescu C. Sia I. Lahr B. D. Hanssen A. D. (2007). Culture-negative prosthetic joint infection. Clin. Infect. Dis. 45, 1113–1119. doi: 10.1086/522184, PMID: 17918072

[B4] Bozic K. J. Kurtz S. M. Lau E. Ong K. Chiu V. (2010). The epidemiology of revision total knee arthroplasty in the United States. Clin. Orthopaedics. Relat. Res. 468, 45–51. doi: 10.1007/s11999-009-0945-0, PMID: 19554385 PMC2795838

[B5] Cai Y. Ding H. Chen X. Chen Y. Huang C. (2023). Optimization and standardization of mNGS-based procedures for the diagnosis of Mycoplasma periprosthetic joint infection: A novel diagnostic strategy for rare bacterial periprosthetic joint infection. Front. Cell. Infect. Microbiol. 13. doi: 10.3389/fcimb.2023.1089919, PMID: 36936762 PMC10014592

[B6] Chen Y. Huang Z. Fang X. Li W. Yang B. (2020). Diagnosis and treatment of mycoplasmal septic arthritis: a systematic review. Int. Orthopaedics. 44, 199–213. doi: 10.1007/s00264-019-04451-6, PMID: 31792575

[B7] Chen S. Kang Y. Li D. Li Z. (2022). Diagnostic performance of metagenomic next-generation sequencing for the detection of pathogens in bronchoalveolar lavage fluid in patients with pulmonary infections: Systematic review and meta-analysis. Int. J. Infect. Dis.: IJID. 122, 867–873. doi: 10.1016/j.ijid.2022.07.054, PMID: 35907477

[B8] Ding H. Huang J. Lin L. Chen Y. Wang Q. (2024). Shedding light on negative cultures in osteoarticular infections: leveraging mNGS to unravel risk factors and microbial profiles. Front. Cell. Infect. Microbiol. 14. doi: 10.3389/fcimb.2024.1457639, PMID: 39654973 PMC11625738

[B9] Fakruddin M. Mannan K. S. Andrews S. (2013). Viable but nonculturable bacteria: food safety and public health perspective. ISRN. Microbiol. 2013, 703813. doi: 10.1155/2013/703813, PMID: 24191231 PMC3804398

[B10] Fang X. Cai Y. Shi T. Huang Z. Zhang C. (2020). Detecting the presence of bacteria in low-volume preoperative aspirated synovial fluid by metagenomic next-generation sequencing. Int. J. Infect. Dis.: IJID. 99, 108–116. doi: 10.1016/j.ijid.2020.07.039, PMID: 32721535

[B11] Fang X. Cai Y. Mei J. Huang Z. Zhang C. (2021). Optimizing culture methods according to preoperative mNGS results can improve joint infection diagnosis. Bone Joint J. 103-b, 39–45. doi: 10.1302/0301-620x.103b1.Bjj-2020-0771.R2, PMID: 33380187

[B12] Forbes B. A. Hall G. S. Miller M. B. Novak S. M. Rowlinson M. C. (2018). Practical guidance for clinical microbiology laboratories: mycobacteria. Clin. Microbiol. Rev. 31, e00038-17. doi: 10.1128/cmr.00038-17, PMID: 29386234 PMC5967691

[B13] Gu W. Miller S. Chiu C. Y. (2019). Clinical metagenomic next-generation sequencing for pathogen detection. Annu. Rev. Pathol. 14, 319–338. doi: 10.1146/annurev-pathmechdis-012418-012751, PMID: 30355154 PMC6345613

[B14] Han D. Diao Z. Lai H. Han Y. Xie J. (2022). Multilaboratory assessment of metagenomic next-generation sequencing for unbiased microbe detection. J. Adv. Res. 38, 213–222. doi: 10.1016/j.jare.2021.09.011, PMID: 35572414 PMC9091723

[B15] Hermsen R. Deris J. B. Hwa T. (2012). On the rapidity of antibiotic resistance evolution facilitated by a concentration gradient. Proc. Natl. Acad. Sci. United. States America 109, 10775–10780. doi: 10.1073/pnas.1117716109, PMID: 22711808 PMC3390829

[B16] Hoffman L. R. Déziel E. D’Argenio D. A. Lépine F. Emerson J. (2006). Selection for Staphylococcus aureus small-colony variants due to growth in the presence of Pseudomonas aeruginosa. Proc. Natl. Acad. Sci. United. States America 103, 19890–19895. doi: 10.1073/pnas.0606756104, PMID: 17172450 PMC1750898

[B17] Illingworth K. D. Mihalko W. M. Parvizi J. Sculco T. McArthur B. (2013). How to minimize infection and thereby maximize patient outcomes in total joint arthroplasty: a multicenter approach: AAOS exhibit selection. J. Bone Joint Surgery. Am. 95, e50. doi: 10.2106/jbjs.L.00596, PMID: 23595076

[B18] Ivy M. I. Thoendel M. J. Jeraldo P. R. Greenwood-Quaintance K. E. Hanssen A. D. (2018). Direct detection and identification of prosthetic joint infection pathogens in synovial fluid by metagenomic shotgun sequencing. J. Clin. Microbiol. 56, e00402-18. doi: 10.1128/jcm.00402-18, PMID: 29848568 PMC6113468

[B19] Kalbian I. Park J. W. Goswami K. Lee Y. K. Parvizi J. (2020). Culture-negative periprosthetic joint infection: prevalence, aetiology, evaluation, recommendations, and treatment. Int. Orthopaedics. 44, 1255–1261. doi: 10.1007/s00264-020-04627-5, PMID: 32449042

[B20] Kim S. J. Cho Y. J. (2021). Current guideline for diagnosis of periprosthetic joint infection: A review article. Hip. Pelvis. 33, 11–17. doi: 10.5371/hp.2021.33.1.11, PMID: 33748021 PMC7952269

[B21] Kurtz S. M. Ong K. L. Lau E. Bozic K. J. Berry D. (2010). Prosthetic joint infection risk after TKA in the Medicare population. Clin. Orthopaedics. Relat. Res. 468, 52–56. doi: 10.1007/s11999-009-1013-5, PMID: 19669386 PMC2795807

[B22] Liu H. Zhang Y. Yang J. Liu Y. Chen J. (2022). Application of mNGS in the etiological analysis of lower respiratory tract infections and the prediction of drug resistance. Microbiol. Spectr. 10, e0250221. doi: 10.1128/spectrum.02502-21, PMID: 35171007 PMC8849087

[B23] Mei J. Hu H. Zhu S. Ding H. Huang Z. (2023). Diagnostic role of mNGS in polymicrobial periprosthetic joint infection. J. Clin. Med. 12, 1838. doi: 10.3390/jcm12051838, PMID: 36902625 PMC10003677

[B24] Mouraviev V. McDonald M. (2018). An implementation of next generation sequencing for prevention and diagnosis of urinary tract infection in urology. Can. J. Urol. 25, 9349–9356., PMID: 29900824

[B25] O’Flaherty B. M. Li Y. Tao Y. Paden C. R. Queen K. (2018). Comprehensive viral enrichment enables sensitive respiratory virus genomic identification and analysis by next generation sequencing. Genome Res. 28, 869–877. doi: 10.1101/gr.226316.117, PMID: 29703817 PMC5991510

[B26] Parvizi J. Erkocak O. F. Della Valle C. J. (2014). Culture-negative periprosthetic joint infection. J. Bone Joint Surgery. Am. volume 96, 430–436. doi: 10.2106/jbjs.L.01793, PMID: 24599206

[B27] Parvizi J. Zmistowski B. Berbari E. F. Bauer T. W. Springer B. D. (2011). New definition for periprosthetic joint infection: from the Workgroup of the Musculoskeletal Infection Society. Clin. Orthopaedics. Relat. Res. 469, 2992–2994. doi: 10.1007/s11999-011-2102-9, PMID: 21938532 PMC3183178

[B28] Parvizi J. Ghanem E. Menashe S. Barrack R. L. Bauer T. W. (2006). Periprosthetic infection: what are the diagnostic challenges? J. Bone Joint Surgery. Am. 88 Suppl 4, 138–147. doi: 10.2106/jbjs.F.00609, PMID: 17142443

[B29] Pulliam L. Porschen R. K. Hadley W. K. (1980). Biochemical properties of CO2-dependent streptococci. J. Clin. Microbiol. 12, 27–31. doi: 10.1128/jcm.12.1.27-31.1980, PMID: 6775006 PMC273513

[B30] Rimoldi S. G. Brioschi D. Curreli D. Salari F. Pagani C. (2023). Traditional cultures versus next generation sequencing for suspected orthopedic infection: experience gained from a reference centre. Antibiot. (Basel Switzerland). 12, 1588. doi: 10.3390/antibiotics12111588, PMID: 37998790 PMC10668678

[B31] Schwarz E. M. Parvizi J. Gehrke T. Aiyer A. Battenberg A. (2019). *20*18 international consensus meeting on musculoskeletal infection: research priorities from the general assembly questions. J. Orthopaedic. Res. 37, 997–1006. doi: 10.1002/jor.24293, PMID: 30977537

[B32] Shi T. Chen H. Liu Y. Wu Y. Lin F. (2024). Clinical applications of metagenomic next-generation sequencing in the identification of pathogens in periprosthetic joint infections: a retrospective study. J. Orthopaedic. Surg. Res. 19, 301. doi: 10.1186/s13018-024-04745-5, PMID: 38760817 PMC11102132

[B33] Tan T. L. Kheir M. M. Shohat N. Tan D. D. Kheir M. (2018). Culture-negative periprosthetic joint infection: an update on what to expect. JB. JS. Open Access 3, e0060. doi: 10.2106/jbjs.Oa.17.00060, PMID: 30533595 PMC6242327

[B34] Thoendel M. Jeraldo P. Greenwood-Quaintance K. E. Chia N. Abdel M. P. (2017). A novel prosthetic joint infection pathogen, mycoplasma salivarium, identified by metagenomic shotgun sequencing. Clin. Infect. Dis. 65, 332–335. doi: 10.1093/cid/cix296, PMID: 28379472 PMC5848249

[B35] Wang C. Huang Z. Li W. Fang X. Zhang W. (2020). Can metagenomic next-generation sequencing identify the pathogens responsible for culture-negative prosthetic joint infection? BMC Infect. Dis. 20, 253. doi: 10.1186/s12879-020-04955-2, PMID: 32228597 PMC7106575

[B36] Wang H. Ren D. Li H. Wang S. (2021). Periprosthetic joint infection caused by mycoplasma hominis, diagnosed using metagenomic sequencing. Int. J. Gen. Med. 14, 7003–7006. doi: 10.2147/ijgm.S330924, PMID: 34707391 PMC8544117

[B37] Weng L. T. Yang D. Q. Chen L. (2022). Materials for selective inhibition of streptococcus mutans and progress in relevant research. Sichuan. da. xue. xue. bao. Yi. xue. ban. = J. Sichuan. Univ. Med. Sci. Edition. 53, 922–928. doi: 10.12182/20220960202, PMID: 36224698 PMC10408796

[B38] Xie Y. Dai B. Zhou X. Liu H. Wu W. (2023). Diagnostic value of metagenomic next-generation sequencing for multi-pathogenic pneumonia in HIV-infected patients. Infect. Drug Resist. 16, 607–618. doi: 10.2147/idr.S394265, PMID: 36733920 PMC9888013

[B39] Yang J. Parvizi J. Hansen E. N. Culvern C. N. Segreti J. C. (2020). *20*20 Mark Coventry Award: Microorganism-directed oral antibiotics reduce the rate of failure due to further infection after two-stage revision hip or knee arthroplasty for chronic infection: a multicentre randomized controlled trial at a minimum of two years. Bone Joint J. 102-b, 3–9. doi: 10.1302/0301-620x.102b6.Bjj-2019-1596.R1, PMID: 32475278

