# Supplementary material for: Microbial culture vs. mNGS: diagnostic variations in periprosthetic joint infection
Source: Front Cell Infect Microbiol. 2025 Oct 28;15:1611332. doi: 10.3389/fcimb.2025.1611332 (PMC12602205; doi:10.3389/fcimb.2025.1611332)
Supplement: Supplementary file 1 [file Table1.docx]

**Supplementary Table 1. The proportion of discrepant results between microbial culture and mNGS testing in PJI group and AF group.**

|  | PJI group (n=122) | |  | AF group (n=45) | |
| --- | --- | --- | --- | --- | --- |
|  | Culture positive | Culture negative |  | Culture positive | Culture negative |
| mNGS positive | 63（51.6%） | 38（31.1%） |  | 4（8.9%） | 0（0%） |
| mNGS negative | 13（10.7%） | 8（6.6%） |  | 1（2.2%） | 40（88.9%） |
